# The future poleward shift of Southern Hemisphere summer mid-latitude storm tracks stems from ocean coupling

**DOI:** 10.1038/s41467-022-29392-4

**Published:** 2022-04-01

**Authors:** Rei Chemke

**Affiliations:** Department of Earth and Planetary Sciences, Weizmann Institute of Science, Rehovot, Israel

**Keywords:** Atmospheric dynamics, Climate change

## Abstract

The latitudinal position of mid-latitude storm tracks has large climate impacts affecting the distribution of precipitation, temperature, humidity, and winds over the extratropics. By the end of this century, climate models project a poleward shift of summer mid-latitude storm tracks in the Southern Hemisphere. Most previous mechanisms for the poleward shift of the storm tracks focused on the role of atmospheric temperature changes. However, the relative roles of other climate system components in the projected storm tracks’ shift have not been examined to date. Here it is shown that thermodynamic ocean coupling is responsible for the future poleward shift of the storm tracks as it overcomes the effect of dynamic ocean coupling to shift the storm tracks equatorward. These results stress the importance of using full-physics ocean models to investigate the future shift of the storm tracks, and of better monitoring ocean coupling processes to improve our preparedness for future climate changes.

## Abstract (web summary)

Mid-latitude storm tracks control most of the weather and climate variability in the extratropics. Here the authors show that, in the Southern Hemisphere, ocean processes will act to shift the summer storm tracks poleward in coming decades.

## Introduction

The weather and climate in the extratropics are primarily set by mid-latitude storms, as they modulate precipitation, humidity, temperature, and winds on daily to multi-decadal timescales. It is thus critical to investigate the future changes in mid-latitude storms to better assess their climate impacts in the coming decades. By the end of this century, climate models project an overall intensification of winter storms in the Southern Hemisphere and over the downstream region of the North Atlantic storm track^1–6^ (although the number of intense cyclones is projected to weaken in the Northern Hemisphere, mostly over the North Atlantic^7,8^). On the other hand, summer storms are projected to weaken in the Northern Hemisphere and to shift poleward in the Southern Hemisphere^1–5^. The latter will have large climate impacts not only at mid-latitudes, but also at high latitudes and in the subtropics; a poleward shift of the storm tracks will increase the storm activity at high latitudes, and reduce it in the subtropics. Several studies have documented a poleward shift of the storms over recent decades^9,10^, however, this recent shift is likely attributed to both stratospheric ozone depletion and an increase in greenhouse gas concentrations^11^. Although the ongoing recovery of the ozone hole is expected to oppose the poleward shift of the storms in the early 21st century^12,13^, the unabated emission of greenhouse gases into the atmosphere is projected to shift the storms poleward over the 21st century.

Different mechanisms were argued to explain the poleward shift of the storm tracks. Since mid-latitude storm tracks arise from baroclinic instability, previous studies suggested that a shift in baroclinicity via changes in both the meridional and vertical temperature gradients^1,4,9,14–18^ (a poleward decline in temperature in a stably stratified fluid give rise to baroclinic instability^19^), as well as changes in the tropopause height^20^, and in the eddy phase speed^21–23^, might be responsible for shifting the storm tracks. In addition, future storms were found to propagate further poleward from their source latitudes, which leads to an additional shift of the storms^24^.

Most above mechanisms have focused on the direct role of the atmospheric temperature response to anthropogenic emissions in modulating the storm tracks’ position. However, other climate components may also play an important role in the future position of the storm tracks, by affecting the atmosphere’s mean state. For example, the use of fixed sea surface temperature in many of the above studies^14–16,18,20^, where the effects of active ocean and sea-ice were missing, prevented an accurate assessment of the mechanism underlying the poleward shift of the storms.

Indeed, ocean coupling processes were also argued to modulate the position of mid-latitude storm tracks (and the position of the mid-latitude jet^25^). Previous idealized model simulations, using atmosphere-only runs, found a poleward shift of the storm tracks in response to increasing the global mean sea surface temperature^14,16,18,26^; surface warming was argued to shift the storm tracks poleward by warming the upper level tropical troposphere (via the moist adiabatic lapse rate), and shifting the baroclinicity poleward^14–17^. In addition, changes in the meridional surface temperature gradient were also argued to affect the position of the storm tracks^18,26^. The use of idealized simulations (atmosphere-only runs with fixed surface temperature, i.e., inactive ocean) and forcings (idealized changes in the meridional gradient or global mean sea surface temperature) has prevented the above studies from quantifying and investigating the relative role of ocean coupling in the projected shift of the storm tracks by the end of this century.

The aim of this work is thus to examine and quantify the role of ocean coupling in shifting mid-latitude summer storm tracks in the Southern Hemisphere (i.e., during December–February (DJF)) over the 21st century. The focus is on the summer in the Southern Hemisphere, since, unlike in the Northern Hemisphere or other seasons, summer storm tracks in the Southern Hemisphere exhibit a robust shift in climate model projections by the end of this century^3–5^. I here follow the previous studies^27–30^ and construct a hierarchy of ocean coupling experiments, which, unlike previous idealized simulations, uses a state-of-the-art fully coupled general circulation model forced under the 20th and 21st century forcings. Such hierarchy, thus, allows isolating and investigating the effect of ocean coupling, and its thermodynamic (i.e., ocean-atmosphere and ocean-sea-ice heat fluxes, SHF) and dynamic (i.e., ocean heat flux convergence, OHFC) components, in the recent and projected poleward shift of mid-latitude storm tracks. This decomposition of ocean coupling also allows one to assess how reliable are models that lack time varying OHFC (e.g., slab ocean models) in investigating the shift of the mid-latitude flow.

## Results

### Quantifying the role of ocean coupling in the poleward shift of mid-latitude storm tracks

**Fig. 1:**
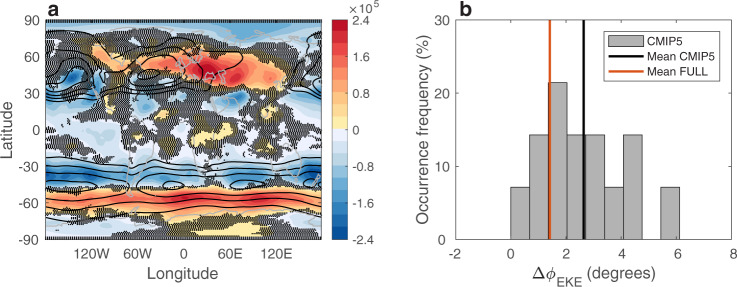
The future poleward shift of mid-latitude eddy kinetic energy. **a** The response to anthropogenic emissions of December–February eddy kinetic energy (EKE, Jm^−2^, colors) in the Coupled Model Intercomparison Project Phase 5 (CMIP5) mean. Black contours show the EKE distribution averaged over the 1980–1999 period in intervals of 3 × 10^5^ Jm^−2^ and maximum values of 1.6 × 10^6^ Jm^−2^ and 2.4 × 10^6^ Jm^−2^ in the Southern and Northern Hemispheres, respectively. Black dots show where less then two thirds of the models agree on the sign of the change. **b** The occurrence frequency (in percentage) of the poleward shift of Southern Hemisphere mid-latitude EKE (Δ*ϕ*_EKE_, degrees) across CMIP5 models (gray bars). Black and red vertical lines show the mean of the CMIP5 and FULL ensembles.

I first demonstrate the future poleward shift of mid-latitude storm tracks across 14 models that participated in the Phase 5 of the Coupled Model Intercomparison Project (CMIP5^31^), and are forced under the Historical (through 2005) and the Representative Concentration Pathway 8.5 forcings (RCP8.5, through 2100, “Methods”). Fig. 1a shows the multi-model mean response to anthropogenic emissions (difference between the last 20 years of the 21st and 20th centuries) of the distribution of DJF transient eddy kinetic energy (EKE, which is a widely used metric for mid-latitude storm tracks^3,32–35^, “Methods”). As was shown in previous studies^1,3–5,17^, in DJF, Southern Hemisphere mid-latitude EKE is projected to weaken on its equatorward flank (blue colors), and to intensify on its poleward flank (red colors), which is suggestive of a poleward shift (in Fig. 1a black contours show the EKE averaged over the 1980–1999 period). To quantify the EKE shift, the change in the latitudinal position of the maximum value of the zonal mean EKE (Δ*ϕ*_EKE_, “Methods”) is calculated across CMIP5 models (Fig. 1b). By the end of this century, in the multi-model mean, mid-latitude EKE is projected to shift poleward by ~2. 6^∘^ (black line), with one standard deviation of ±1.47^∘^ across the models (gray bars). Note that CMIP5 models adequately capture the recent (over the 1979–2019 period) observed trend in *ϕ*_EKE_; the recent shift of mid-latitude storm tracks from two reanalyses (JRA55 and Era-Interim) fall well within the shift in CMIP5 models (Supplementary Fig. 1a). This suggests that climate models can be used to investigate the future shift of the storm tracks.

To isolate and quantify the role of ocean coupling in the future poleward shift of mid-latitude EKE, I follow previous studies^27–30^ and next analyze a hierarchy of ocean coupling experiments in three large ensembles of model simulations (Methods). All ensembles are conducted using the Community Earth System Model (CESM1^36^), forced with the same Historical and RCP8.5 forcings as in CMIP5, but differ in their ocean coupling processes. The first ensemble^37^, which uses the full configuration of CESM1 (hereafter refer to as FULL), including a full-physics ocean component, comprises any effects of ocean coupling on the response of EKE to anthropogenic emissions. In the second ensemble (hereafter refer to as SOM, which stands for slab ocean model), while thermodynamic coupling is active, dynamic coupling is fixed at the climatological preindustrial values of the fully-coupled model; this is done by replacing only the full-physics ocean with a slab ocean model with fixed OHFC and mixed-layer depth (Methods). Thus, since both ensembles are initialized from a similar preindustrial climatology (Supplementary Fig. 2), comparing the FULL and SOM ensembles isolates the role of dynamic coupling (i.e., changes in both horizontal heat transport and vertical heat uptake by the deep ocean) in the EKE response to external forcings over the 20th and 21st centuries.

In the third ensemble (hereafter refer to as NOM, which stands for no ocean model) both dynamic and thermodynamic ocean coupling are fixed; this is done by fixing the sea surface temperature in the slab ocean model configuration at preindustrial values (note that unlike in atmosphere-only simulations, where both sea surface temperature and sea-ice are prescribed, here only the sea surface temperature is fixed, such that one could isolate only the role of ocean coupling). Thus, while comparing the EKE in the FULL and NOM ensembles allows isolating the role of net ocean coupling in the EKE response, comparing the SOM and NOM ensembles allows isolating the role of thermodynamic coupling (the effects of ocean-atmosphere and ocean-sea-ice heat fluxes on the mixed-layer temperature) (see “Methods” for more details on the ensembles). Note that isolating the role of ocean coupling includes all processes that affect the future position of the storm tracks via changes in the ocean coupling component in question, including any effects that dynamic and thermodynamic ocean coupling may have on each other.

Before quantifying the role of ocean coupling in the EKE response, I first ensure that the EKE shift in the FULL ensemble is not an outlier within the CMIP5 models and that it captures the observed shift in EKE over recent decades. First, the mean of the FULL ensemble not only shows a poleward shift of ~1.4^∘^ (red line in Fig. 1b), which is well within the CMIP5 response, but it also shows a similar distribution of the EKE response as in the CMIP5 mean (Supplementary Fig. 3). Second, similar to CMIP5 models, the FULL ensemble also adequately captures the recent EKE shift; the 1979–2019 trends in *ϕ*_EKE_ from two reanalyses fall well within the spread of the ensemble (Supplementary Fig. 1b). Thus, the CESM1 can be used for investigating the role of ocean coupling in the poleward shift of the EKE.

**Fig. 2:**
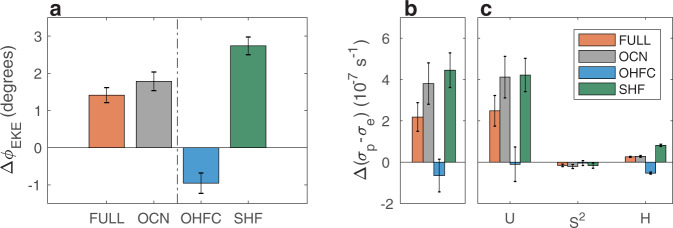
The role of ocean coupling in the poleward shift of mid-latitude eddy kinetic energy. **a** The projected shift in eddy kinetic energy (Δ*ϕ*_EKE_, degrees) in the mean of the FULL ensemble (red bar), and the relative contribution to the shift of the EKE from ocean coupling (gray bar, difference between FULL and NOM), and from decomposing the ocean coupling to dynamic coupling (OHFC, blue bar, difference between FULL and SOM) and thermodynamic coupling (SHF, green bar, difference between SOM and NOM). **b** The response to anthropogenic emissions of the growth rate on the poleward flank of the EKE, relative to the equatorward flank (Δ(*σ*_*p*_ − *σ*_*e*_), 10^−7^ s^−1^). **c** The relative contribution to the growth rate response from the mean zonal wind (*u*), static stability (*S*^2^) and tropopause height (*H*) in the FULL ensemble (red bars), and the contributions from ocean coupling (gray bars), thermodynamic coupling (SHF, green bars) and dynamic coupling (OHFC, blue bars). The error bars show the 95% confidence interval based on a Student’s t-distribution.

Similar to the findings on the poleward shift of the zonal wind^25^, isolating the role of ocean coupling in CESM1 (i.e., the difference between FULL and NOM, gray bar in Fig. 2a) reveals that having an active ocean accounts for nearly all of the EKE shift (compare red and gray bars in Fig. 2a); ocean coupling results in a poleward shift of ~1.78^∘^. Thus, without an active ocean the EKE is not projected to shift poleward over the 21st century. To qualitatively ensure that the role of ocean coupling to shift the EKE poleward is not dependent on the specific configuration of the CESM1, the EKE shift is next analyzed in a different model.

In particular, the shift is analyzed in the fully-coupled and atmosphere-only configurations of the IPSL-CM6A-LR, in response to quadrupling of CO_2_ concentrations (“Methods”; an atmosphere-only/fixed sea surface temperature ensemble is not available in other models). Since, under the RCP8.5 scenario, CO_2_ concentrations are expected to approximately quadruple by the end of this century, the idealized abrupt 4 × CO_2_ experiment allows to qualitatively assess the role of ocean coupling in shifting the EKE poleward. Unlike in the NOM ensemble, where only the sea surface temperature is fixed at preindustrial values, here both sea surface temperature and sea-ice are fixed at preindustrial values. Thus, comparing the fully-coupled and atmosphere-only models isolates the role of both ocean and sea-ice coupling in the EKE shift. Similar to CESM1, without an active ocean, the EKE in IPSL-CM6A-LR is not expected to shift poleward in response to quadrupling of CO_2_ concentrations; changes in ocean/sea-ice account for nearly all of the EKE shift (Supplementary Fig. 4). Note that the different forcings (RCP8.5 vs. 4 × CO_2_) and sea-ice coupling are not likely to qualitatively affect the above results, as the important role of ocean coupling to shift the storm tracks poleward is evident also using the fully-coupled and atmosphere-only configurations of CESM2 (the CMIP6 version of CESM) under quadrupling of CO_2_ concentrations (Supplementary Fig. 4). Thus, the effect of ocean coupling to shift the EKE poleward is likely not an artifact of the CESM1 model.

Decomposing the effect of ocean coupling to dynamic (blue bar in Fig. 2a, OHFC, i.e., the difference between FULL and SOM) and thermodynamic (green bar, SHF, i.e., the difference between SOM and NOM) ocean coupling shows that OHFC acts to shift the EKE equatorward by ~0.95^∘^, and thus to reduce the future poleward shift by ~40%. In contrast, thermodynamic coupling is responsible for the poleward shift of the storm tracks, as it acts to shift the EKE poleward by ~2.74^∘^. The important roles of both thermodynamic and dynamic coupling in the future position of the storm tracks emphasizes that it is imperative to use fully-coupled models, which include both dynamic and thermodynamic coupling processes, to investigate the shift of mid-latitude summer storm tracks in the Southern Hemisphere. In particular, fully-coupled models should be used to accurately assess the importance of previously suggested atmospheric components in shifting the storm tracks poleward (e.g., changes in tropopause height and meridional and vertical atmospheric temperature gradients^14–16,20^), which were found using fixed sea surface temperature models.

### Investigating the mechanism underlying the effects of ocean coupling on the poleward shift of mid-latitude EKE

I now turn to investigate the role of ocean coupling in the poleward shift of EKE. First, I follow previous studies^35,38,39^, and conduct a linear normal mode instability analysis to the quasigeostrophic equations (a simple set of equations that describe the mid-latitude flow), linearized about a time and zonal mean state (“Methods”). Such analysis allows one to investigate the growth rate of the eddies, which is a widely used metric for the baroclinicity of the flow^1,5,17,35,39^ (recall that mid-latitude transient eddies are driven by baroclinic instability). In particular, using the zonal mean zonal wind, static stability, and tropopause height, averaged over the last 20 years of either the 20th or 21st centuries, the maximum growth rate is calculated at each latitude over the equatorward and poleward flanks of the 20th century EKE (each flank is defined as 12.5^∘^ poleward/equatorward of *ϕ*_EKE_). The response (i.e., the difference between the last 20 years of the 21st and 20th centuries) of the difference in the growth rate between the poleward and equatorward flanks (Δ(*σ*_*p*_ − *σ*_*e*_)) yields the changes in baroclinicity associated with the EKE shift: positive values indicate of a poleward shift in baroclinicity, and negative values an equatorward shift in baroclinicity.

The resulting changes in the growth rate capture the behaviour of the EKE shift across the ensembles (Fig. 2b). First, as noted in the previous studies^1,17^, in the FULL ensemble, the growth rate increases more on the poleward flank of the storm tracks, relative to the equatorward flank (by ~2.2 × 10^−7^ s^−1^, red bar), which implies a poleward shift in baroclinicity, and thus of EKE. Second, as with the EKE shift, also here ocean coupling is responsible for the shift in the growth rate (by ~3.8 × 10^−7^ s^−1^, gray bar), via changes in thermodynamic coupling (~4.4 × 10^−7^ s^−1^, green bar), while dynamic coupling acts to reduce the shift in the growth rate (~−6.4 × 10^−8^ s^−1^, blue bar). Thus, further investigating the changes in the growth rate can provide a better understanding on how ocean coupling affects the EKE shift.

The relative simplicity of the linear normal mode instability analysis allows one to investigate the contribution of each of the input parameters (i.e., zonal mean zonal wind, static stability, and tropopause height) to shift the growth rate poleward. In particular, the 21st century growth rate is recalculated using the 21st century value of only one of the mean fields, while all other mean fields are fixed at their 20th century values; the resulting response (i.e., the difference between the 21st and 20th centuries) yields the contribution of each of the mean fields to changes in the growth rate. Note, the sum of the relative contribution from each mean field adequately captures the growth rate of the 21st century (Supplementary Fig. 5), and thus such decomposition can be used to isolate the effects of the mean fields on the growth rate changes.

Similar to previous studies^1,17^, in the FULL ensemble, changes in the zonal wind (*U*, red bars in Fig. 2c) are responsible for most of the changes in the growth rate. Since the EKE shift stems from ocean coupling, and in particular from thermodynamic coupling (Fig. 2a), the latter also shifts the growth rate poleward by modulating the zonal wind (with a small contribution from tropopause height changes) (gray and green bars in Fig. 2c). In contrast, the equatorward shift of the growth rate by dynamic coupling is mostly due to changes in the tropopause height^20^ (*H*, blue bars in Fig. 2c). Static stability (*S*^2^) is found to have a minor effect on the growth rate changes. I, thus, next investigate the effect of thermodynamic coupling on the zonal wind, and of dynamic coupling on the tropopause height. Note that the larger effect of ocean coupling, and in particular of thermodynamic coupling, on the poleward shift in the growth rate, relative to its effect on the shift in EKE (compare red and gray bars in Fig. 2a and b) suggests that the instability analysis might be more sensitive to changes in the zonal wind, than changes in static stability.

**Fig. 3:**
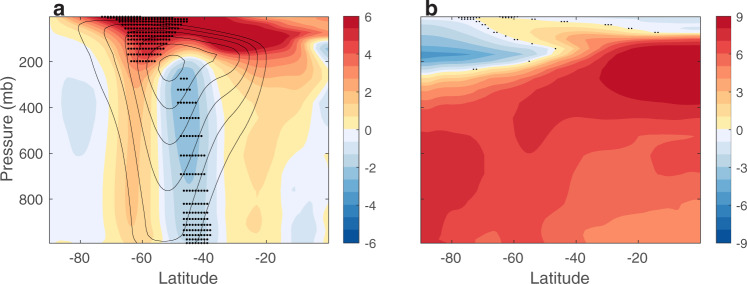
The contribution of thermodynamic coupling to the response of the zonal mean flow. The effect of thermodynamic coupling on the response of the zonal mean **a** zonal wind (ms^−1^) and **b** temperature (K) to anthropogenic emissions. Black contours show the mean zonal wind averaged over the 1980–1999 period from the FULL ensemble in intervals of 5 ms^−1^ and maximum value of 30 ms^−1^. Small black dots in panel a show where the zonal wind changes are linked to the EKE shift (i.e., with *R*^2^ ≥ 0.85). Black dots in panel **b** shows where the response is not statistically significant at the 5% level based on a Student’s t-test.

The contribution of thermodynamic coupling to the response of the mean zonal wind is shown in Fig. 3a (colors; black contours shows the mean zonal wind averaged over the last 20 years of the 20th century from the FULL ensemble). Thermodynamic coupling acts to intensify the zonal wind on its poleward and equatorward flanks, mostly at upper levels, and to reduce its intensity at the jet’s core. To explore which thermodynamically induced zonal wind changes are linked to the shift in EKE, I regress, at each location, the time series of the effect of thermodynamic coupling on the zonal wind changes (i.e., the time series of each point in Fig. 3a) on the time series of the effect of thermodynamic coupling on the latitudinal position of EKE. The small black dots in Fig. 3a show regions where the thermodynamically induced changes in the zonal wind are strongly associated with the EKE shift (i.e., regions where the regression yields *R*^2^ ≥ 0.85). The EKE shift is mostly linked to the increase in the vertical shear of the zonal wind; the EKE is associated with the increase in the zonal wind at upper levels over the poleward flank of the jet, and the reduction in zonal wind at lower levels at the jet’s core.

**Fig. 4:**
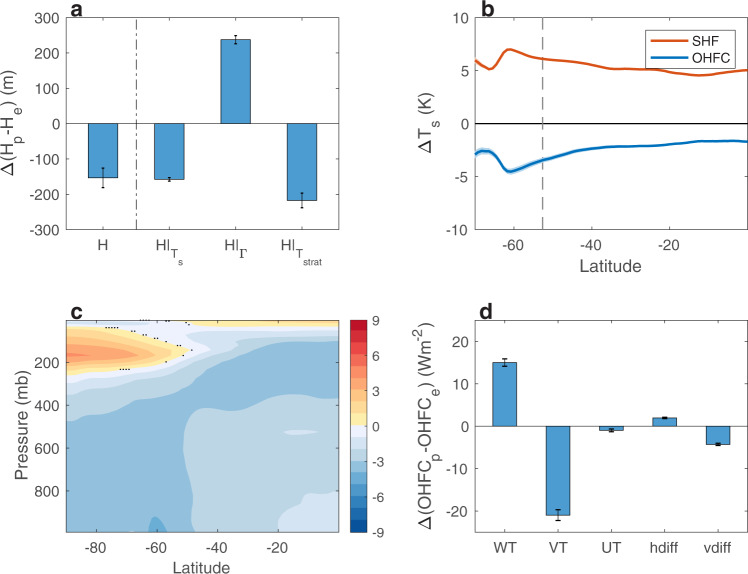
The contribution of dynamic coupling to the response of the tropopause height. **a** The effect of dynamic coupling on the response to anthropogenic emissions of the tropopause height on the poleward flank of the eddy kinetic energy (EKE), relative to the equatorward flank (Δ(*H*_*p*_ − *H*_*e*_),  m), and the relative contributions to Δ(*H*_*p*_ − *H*_*e*_) from surface temperature ($H{| }_{{T}_{s}}$), tropospheric lapse rate (*H*∣_Γ_) and stratospheric temperature ($H{| }_{{T}_{{{{{{{{\rm{strat}}}}}}}}}}$). **b** the effect of thermodynamic (red) and dynamic (blue) coupling on the response of the surface temperature (K) to anthropogenic emissions. Shading shows two standard deviations across the ensemble members, and the vertical dashed line shows the climatological position of the storm tracks. **c** The effect of dynamic coupling on the response of atmospheric temperature (K) to anthropogenic emissions. Black dots show where the response is not statistically significant at the 5% level based on a Student’s t-test. **d** The response to anthropogenic emissions of the different components of mixed-layer ocean heat flux convergence (OHFC) on the poleward flank of the EKE, relative to the equatorward flank (Δ(OHFC_*p*_ − OHFC_*e*_), Wm^−2^); vertical (*WT*), meridional (*VT*) and zonal (*UT*) heat flux, and horizontal (hdiff) and vertical (vdiff) heat diffusion. The error bars show the 95% confidence interval based on a Student’s t-distribution.

To better understand how thermodynamic coupling modulates the vertical shear of the zonal wind, and thus the EKE position, I next explore the effects of thermodynamic coupling on the meridional temperature gradient, which is in balance with the zonal wind shear. Thermodynamic coupling accounts for most of the global warming temperature pattern (Fig. 3b): by warming the surface through all latitudes (red line in Fig. 4b) thermodynamic coupling produces the upper tropical tropospheric warming (by modulating the moist adiabatic lapse rate) and lower stratospheric cooling at high latitudes. These temperature changes increase the upper-level meridional temperature gradient (together with the zonal wind shear), on the poleward flank of the jet, which pushes the baroclinicity and thus the EKE poleward; similar effect of global surface warming was found in atmosphere-only runs^14^. This result adds to previous findings on the importance of upper level changes in baroclinicity in shifting the mid-latitude storm tracks^1,4,9,14,15,17^.

I next investigate the effect of dynamic coupling on the different responses of the tropopause height on the poleward and equatorward flanks of the EKE (Δ(*H*_*p*_ − *H*_*e*_)). The tropopause height is defined, following the WMO, as the lowest level where the lapse rate reaches 2 Kkm^−1^, and stays, on average, below 2 Kkm^−1^ in all higher levels within 2 km^40^. OHFC acts to reduce the tropopause height more on the poleward flank of the EKE than on the equatorward flank by ~150 m (leftmost bar in Fig. 4a). This leads to a larger reduction of the growth rate on the poleward flank, which thus acts to shift the EKE equatorward (Fig. 2).

To further reveal the effects of dynamic coupling on the tropopause height, I follow previous studies^41–43^, and analyze a simple equation for the changes in tropopause height. Assuming a constant tropospheric lapse rate (Γ), and isothermal stratosphere, the response of the tropopause height to anthropogenic emissions can be written as follows, ${{\Delta }}H=-\frac{{{\Delta }}{T}_{s}}{{{\Gamma }}}-\frac{H{{\Delta }}\Gamma }{{{\Gamma }}}+\frac{{{\Delta }}{T}_{{{{{{{{\rm{strat}}}}}}}}}}{{{\Gamma }}}$, where the righthand side terms account for the relative contribution to the tropopause height changes from surface temperature (*T*_*s*_), tropospheric lapse rate, and stratospheric temperature (*T*_strat_), respectively. Note that the sum of the relative contributions of the righthand side terms account for ~90% of Δ(*H*_*p*_ − *H*_*e*_). Thus, the above equation is adequate to investigate the effects of OHFC on the tropopause height.

The terms that are responsible for the effect of OHFC to reduce the tropopause height more on the poleward flank, than on the equatorward flank of the EKE, are the surface and stratospheric temperature responses (${\left.H\right|}_{{T}_{s}}$ and ${\left.H\right|}_{{T}_{{{{{{{{\rm{strat}}}}}}}}}}$, Fig. 4a). The contributions of OHFC to changes in surface and atmospheric temperatures are shown in Fig. 4b, c, respectively. First, by 2100, OHFC acts to cool the surface temperature more on the poleward flank of the EKE than the equatorward flank (see blue line around the vertical dashed line in Fig. 4b), yielding a larger reduction of the tropopause height on the poleward flank. To explore which changes in OHFC are associated with cooling the surface over the poleward flank of the EKE more than over the equatorward flank, I next analyze the responses of the different OHFC components in the mixed-layer, on the poleward and equatorward flanks of the EKE (Δ(OHFC_*p*_ − OHFC_*e*_) in Fig. 4d). The term that mostly contributes to the larger surface cooling by OHFC on the poleward flank of the storm tracks, relative to the equatorward flank, is the meridional heat flux (VT), which suggests an enhanced northward heat transport across the storm tracks’ core. All other terms either result in a larger warming on the poleward flank (vertical heat flux, WT), or have a minor effect on the different surface temperature response in the two flanks (zonal heat flux, and diffusion processes). Second, unlike thermodynamic coupling, which has a warming effect on the surface (red line in Fig. 4b), and thus acts to cool the lower stratosphere at high latitudes (Fig. 3b), the surface cooling effect of dynamic coupling (a result of increased oceanic heat uptake by the deep ocean; blue line in Fig. 4b) acts to warm the lower polar stratosphere (Fig. 4c), thus reduce the tropopause height more on the poleward flank of the EKE. The above effects of vertical and meridional OHFC on the tropopause height, via stratospheric and surface temperatures, act to shift the baroclinicity, and thus the EKE equatorward (Fig. 2).

**Fig. 5:**
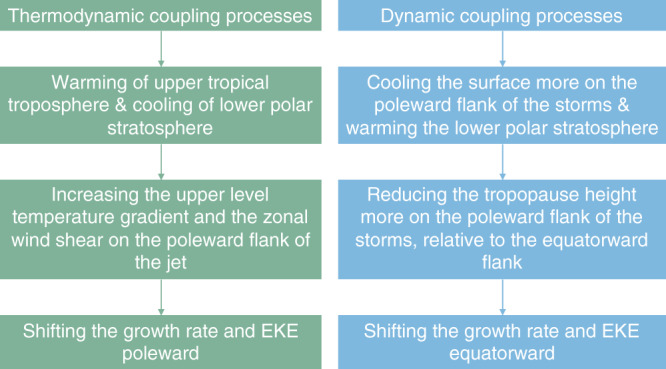
Summary of the mechanisms underlying the latitudinal shift of mid-latitude eddy kinetic energy via ocean coupling processes. The flowcharts show via which processes thermodynamic (in green) and dynamic (in blue) ocean coupling affect the position of mid-latitude storm tracks.

It is interesting that although dynamic and thermodynamic coupling yield similar patterns of temperature changes, only with opposite signs (compare Figs. 3b and Fig. 4c and blue and red lines in Fig. 4b), their effects on the shift of the storm tracks stem mostly from different processes, i.e., changes in upper level wind shear vs. changes in tropopause height (see also Fig. 5 for a summary of the proposed mechanisms discussed above). This might occur since the magnitude of the temperature changes via dynamic and thermodynamic coupling are different, especially at low latitudes. Thermodynamic coupling considerably warms the surface at both low and high latitudes (Fig. 4b), and thus acts both to considerably warm the upper tropical troposphere and cool the lower polar stratosphere. These temperature changes enhance the upper level temperature gradient, which increases the baroclinicity at high latitudes and pushes the storm tracks poleward. In contrast, the surface cooling due to dynamic coupling is overall weaker, especially at low latitudes. As a result, in upper levels, dynamic coupling affects mostly the lower polar stratospheric temperatures, and has a relative small effect on the upper tropical tropospheric temperatures, and thus also on the upper level temperature gradient (while in some members dynamic coupling acts to shift the storm tracks equatorward also via zonal wind changes, in other members the effect of zonal wind changes is minor and even opposite; error bars in Fig. 2c). Furthermore, the stronger surface cooling effect of dynamic coupling at high latitudes leads to a larger reduction in the tropopause height at high latitudes, relative to low latitudes, which pushes the baroclinicity and the storm tracks equatorward.

## Discussion

Given the large climate impacts of the poleward shift of mid-latitude storm tracks it is critical to improve our understanding on ocean coupling processes, as they are found here to control the future position of the storm tracks. In particular, the large effects of both dynamic and thermodynamic coupling on the future position of the storm tracks highlight the importance of analyzing the storm tracks’ shift using fully-coupled models, where both dynamic and thermodynamic ocean coupling are present, rather than using slab-ocean models. Furthermore, while climate models adequately capture the poleward shift of mid-latitude storm tracks over recent decades (Supplementary Fig. 1), their inability to simulate the recent cooling of the Southern Ocean surface^44,45^ (and the associated changes in air-sea heat fluxes^46,47^), which was shown to affect the simulated recent changes in mid-latitude eddy heat fluxes^48^, might also hinder the models’ ability to accurately simulate the future shift of the storm tracks. Thus, better monitoring ocean-atmosphere heat fluxes (which are responsible for the future poleward shift of the storm tracks) and the Southern Ocean dynamics (which nearly halves the future poleward shift of the storm tracks), and improving their parameterizations in climate models, would allow us not only to better assess the future changes in the storm tracks, but also to improve climate change adaption strategies.

## Methods

### EKE

To investigate the poleward shift of mid-latitude storm tracks I follow the previous studies^3,32–35^ and calculate the DJF vertically integrated kinetic energy of transient eddies (EKE), ${{{{{{{\rm{EKE}}}}}}}}=\frac{1}{g}\int\nolimits_{0}^{{p}_{s}}\left(\overline{{u}^{^{\prime} 2}}+\overline{{v}^{^{\prime} 2}}\right){{{{{\rm{d}}}}}}p$, where *g* is gravity, *p*_*s*_ is surface pressure, *p* is pressure, *u* and *v* are the zonal and meridional winds, respectively, and prime denotes deviation from monthly mean (denoted by overbar). I here choose to define the eddies as deviation from monthly mean, since output of daily data is not available in the CESM ensembles (output of monthly data of kinetic energy is available in CESM). Nonetheless, defining the eddies using a band-pass filter of 2.5–6 days^49,50^ or as deviations from monthly mean yields a very similar shift of the storm tracks by the end of this century (of ~2°; compare Fig. 1b and Supplementary Fig. 6). Thus, defining the EKE as deviations from monthly mean mostly captures the behavior of synoptic time-scale phenomena. The position of the storm tracks at mid-latitudes is defined as the latitude of the maximum zonal mean EKE, after applying a 0.1° latitudinal cubic interpolation. Throughout the text, the response to anthropogenic emissions is defined as the difference between the last 20 years of the 21st and 20th centuries.

### CMIP5 models

I analyze the daily output of zonal and meridional winds from 14 models that participate in the Phase 5 of the Coupled Model Intercomparison Project (CMIP5^31^) (listed in Supplementary Table 1); CMIP5 models serve as the scientific basis for the report of the United Nations Intergovernmental Panel on Climate Change (IPCC). All 14 models comprise all components in the climate system (atmosphere, ocean, land, and ice), and run under the same specifically-designed experiments. In particular, I analyze two experiments: Historical, where the models are forced with radiative forcing (consistent with observations) through 2005, and, the Representative Concentration Pathway 8.5 (RCP8.5), which accounts for a high emission scenario through 2100. The use of multiple models to investigate the future changes in the storm tracks is imperative in order to ensure that the results are not dependent on the specific formulation of a single model; while the models are forced with the same forcing agents they differ in their physical or numerical formulations. Lastly, since different modelling centres have created several simulations under the same forcings, which only differ by their realization/initialization/physics, I here follow previous studies and examined only the ’r1i1p1’ simulation in order to weigh all models equally; the different simulations were named in the rip-nomenclature, r for realization, i for initialization and p for physics, followed by an integer.

### Reanalyses

To validate that the models adequately simulate the recent changes in EKE, I compare the recent (1979-2019) EKE trends in models to the EKE trends from two different reanalyses (The ECMWF Era-Interim^51^ and JRA-55^52^). Reanalyses constrain atmospheric models with observations (e.g., rawinsondes, satellite, and surface marine and land data), thus provide the best estimate for the state of the atmosphere in recent decades. The use of models in reanalyses is critical as it allows one to overcome the fact that observations of atmospheric variables are not continuous in space and time.

### Hierarchy of ocean coupling experiments

To examine the effect of ocean coupling, and its thermodynamic and dynamic components, on the poleward shift of the storm tracks I make use of three large ensembles of model simulations using the Community Earth System Model (CESM^37^). The first member in each ensemble is initialized from a multi-century preindustrial control simulation, and run from 1850 to 1920 under the Historical forcing. In 1920, all other members branch of the first member using a very small perturbation to the air temperature (${{{{{{{\mathcal{O}}}}}}}}1{0}^{-14}$), and run from 1920 to 2100 under the same Historical and RCP8.5 forcings as in CMIP5. The use of large ensembles allows one to investigate the forced response of the storm tracks to external forcings in the presence of the background internal climate variability; the mean of each ensemble isolates the forced response of the system, as it averages out the internal climate variability.

The sole difference across the ensembles is ocean coupling, which can be investigated via the mixed-layer temperature equation, $\rho {c}_{p}h\frac{\partial {{{{{{{\rm{T}}}}}}}}}{\partial t}={{{{{{{\rm{SHF}}}}}}}}+{{{{{{{\rm{OHFC}}}}}}}}$, where, *ρ* is sea-water density, *c*_*p*_ is the ocean specific heat capacity, *h* mixed-layer depth, SHF represents the net heat flux into the ocean, from both atmosphere and sea-ice (thermodynamic coupling), and OHFC is dynamic coupling (−∇ ⋅ (*vT*); both horizontal and vertical heat transport). The first ensemble comprises 40 members and uses the full configuration of the CESM1^37^ (FULL). The presence of a full-physics ocean in FULL allows ocean coupling (SHF and OHFC) to affect the poleward shift of the storm tracks over the 20th and 21st centuries. In the second ensemble (SOM), which comprises 20 members, only the fully-physics ocean is replaced with a slab ocean model (all other components are similar to the fully-coupled model) with fixed OHFC and mixed-layer depth (i.e., fixed dynamic coupling), calculated at each location from the climatology of the preindustrial run of the full configuration of CESM1, using the mixed-layer temperature equation^53^. Thus, since in SOM dynamic coupling cannot affect the shift of the storm tracks, the difference in the storm tracks response to anthropogenic emissions between the FULL and SOM ensembles allows isolating and quantifying the role of dynamic coupling (i.e., changes in both horizontal heat transport and vertical heat uptake by the deep ocean) in the poleward shift of mid-latitude storm tracks.

In the third ensemble (NOM), which also comprises 20 members integrated using the slab ocean model of CESM1, the sea surface temperature is fixed at preindustrial values (calculated from the climatology of the preindustrial run of the full configuration of CESM1). Thus, since in NOM there is no active ocean coupling (i.e., both ocean dynamic and thermodynamic coupling are fixed), the difference in the storm tracks response between the FULL and NOM ensembles allows isolating and quantifying the role of ocean coupling in the poleward shift of mid-latitude storm tracks. Furthermore, the difference in the storm tracks response between the SOM and NOM ensembles allows isolating the effect of thermodynamic coupling on the storm tracks’ response (i.e., the impact of ocean-atmosphere and ocean-sea-ice heat fluxes on the mixed-layer temperature). Thus, by construction, the sum of the effect of thermodynamic coupling (the difference between SOM and NOM) and dynamic coupling (the difference between FULL and SOM) yields the net effect of ocean coupling (the difference between FULL and NOM), as inferred from fixed sea surface temperature runs. The isolation of each ocean coupling component includes all of its indirect effects on the future position of the storm tracks via any other climate components.

Note that the use of the fully-coupled model to prescribe the slab ocean model yields similar preindustrial climatology of EKE in the two model configurations (Supplementary Fig. 2); the different ensembles are thus initialized from similar background states, and the different EKE response across the ensembles is only due to the different ocean coupling processes. Lastly, to demonstrate that the ensembles are sufficiently large to capture the internal variability of the shift of the storm tracks, I calculate the variability of the latitudinal position of the storm tracks across different number of ensemble members. Specifically, for each number of ensemble members, *n*, and at each year over the 1920–2100 period, I calculate the standard deviation over all combination of *n* ensemble members (or up to 1000 random combinations), and then average over all combinations and over all years. 98% of the variability of the latitudinal position of the storm tracks in the FULL, SOM, and NOM is already captured using 14, 11, and 9 members in each ensemble, respectively (Supplementary Fig. 7). Thus, the FULL, SOM, and NOM ensembles are sufficiently large to capture the internal variability of the shift of the storm tracks.

### CMIP6 models

To qualitatively validate the key role of ocean coupling to shift the storm tracks poleward in CESM1, and to ensure that it is independent on the specific formulations of the CESM1, I also analyze the poleward shift of the storm tracks in a different model: the IPSL-CM6A-LR^54^. In particular, I investigate the poleward shift of the storm tracks in response to quadrupling of CO_2_ concentrations, relative to preindustrial values, in the fully-coupled and atmosphere-only configurations of IPSL-CM6A-LR. I use this model, as only the IPSL-CM6A-LR (aside from CESM2) has available daily data, needed for calculating the EKE, under the CMIP6 piControl, abrupt-4xCO2, piSST and piSST-4xCO2 experiments. I compare the storm tracks’ response in CESM1 under the RCP8.5 scenario to the storm tracks’ response under quadrupling of CO_2_ concentrations, since by 2100, CO_2_ concentrations are projected to approximately quadruple, relative to preindustrial values, according to the RCP8.5 scenario. I also examine the shift in EKE in CESM2^55^ in the above runs, which allows me to examine the effect of different forcing agents on the role of ocean coupling in shifting the storms.

Lastly, I also examine the shift in EKE under the Historical and SSP5-8.5 experiments (using the r1i1p1f1 member) of the following 20 CMIP6 models: ACCESS-CM2, ACCESS-ESM1-5, BCC-CSM2-MR, CanESM5, CESM2-WACCM, CMCC-CM2-SR5, EC-Earth3, EC-Earth3-Veg, FGOALS-g3, GFDL-CM4, INM-CM4-8, INM-CM5-0, IPSL-CM6A-LR, MIROC6, MPI-ESM1-2-HR, MPI-ESM1-2-LR, MRI-ESM2-0, NorESM2-LM, NorESM2-MM, TaiESM1.

### Linear normal mode instability analysis

Using a linear normal mode instability analysis I investigate by which processes ocean coupling affects the poleward shift of mid-latitude storm tracks. Following previous studies^35,38,39^, I numerically solve the conservation of interior quasigeostrophic potential vorticity (*q*, between the surface, *p*_*s*_, and the tropopause height, *H*_*p*_) and of buoyancy at the vertical boundaries, linearized about the zonal mean state (denoted by overbars),

1 $$\frac{\partial q^{\prime} }{\partial t} +\overline{u}\frac{\partial q^{\prime} }{\partial x}+\frac{\partial \psi ^{\prime} }{\partial x}\frac{\partial \overline{q}}{\partial y}=0\,,{H}_{p} \; < \; p \; < \;{p}_{s}\\ \frac{\partial }{\partial t}\frac{\partial \psi ^{\prime} }{\partial p} +\overline{u}{{\mbox{}}}\frac{\partial }{\partial x}{{\mbox{}}}\frac{\partial \psi ^{\prime} }{\partial p}-\frac{\partial \psi ^{\prime} }{\partial x}\frac{\partial \overline{u}}{\partial p}=0,\,p={H}_{p},{p}_{s},$$

The quasigeostrophic eddy potential vorticity can be written as, $q^{\prime} ={\nabla }^{2}\psi ^{\prime} +{{\Gamma }}\psi ^{\prime}$, where *ψ* = *ϕ*/*f* is the streamfunction, *ϕ* is the geopotential, *f* is the Coriolis parameter, ${{\Gamma }}=\frac{\partial }{\partial p}\frac{{f}^{2}}{{S}^{2}}\frac{\partial }{\partial p}$, ${S}^{2}=-\frac{1}{\overline{\rho }\overline{\theta }}\frac{\partial \overline{\theta }}{\partial p}$ is static stability, *ρ* is density and $\frac{\partial \overline{q}}{\partial y}=\beta -{{\Gamma }}\overline{u}$ is the zonal mean quasigeostrophic potential vorticity gradient, where *β* is the meridional derivative of *f*. The tropopause height is defined, following the WMO, as the lowest level where the lapse rate reaches 2 Kkm^−1^, and stays, on average, below 2 Kkm^−1^ in all higher levels within 2 km^40^.

By assuming a plane wave solution of the form, $\psi ^{\prime} ={{{{{{{\rm{Re}}}}}}}}\left\{\hat{\psi ^{\prime} }(p){{{{{\rm{e}}}}}}^{{{{{{\rm{i}}}}}}(kx-\omega t)}\right\}$, where *k* and *ω* are the zonal wavenumber and frequency, respectively, Eq. 1 can be written as an eigenvalue problem (with *ω* as the eigenvalues). The zonal mean fields (zonal wind, static stability, and tropopause height), averaged over either the 1980–1999 or 2080–2099 periods, are used to solve the resulting eigenvalue problem. The imaginary component of the resulting eigenvalues represents the growth rate of the waves (a widely used measure for the baroclinicity of the flow), and I here analyze the fastest growth rate. Note that unlike simpler metrics for the growth of the waves (e.g., Eady growth rate) or for baroclinicity (e.g., the meridional temperature gradient), the above equation accounts for vertical variations in the zonal wind shear (and thus in the meridional temperature gradient) and static stability, which are important for capturing the future changes in EKE.

## Supplementary information

Supplementary Information

## Data Availability

The data used in the manuscript is publicly available for CMIP5 data (https://esgf-node.llnl.gov/projects/cmip5/), CMIP6 data (https://esgf-node.llnl.gov/projects/cmip6/), Era-Interim (https://www.ecmwf.int/), JRA-55 (https://rda.ucar.edu/) and the fully configuration CESM data (http://www.cesm.ucar.edu/). Due to the large volume of the SOM and NOM ensembles, these are available upon request from rei.chemke@weizmann.ac.il.

## Code availability

Any codes used in the manuscript are available at https://www.weizmann.ac.il/EPS/Chemke/code
