# Supplementary material for: The future poleward shift of Southern Hemisphere summer mid-latitude storm tracks stems from ocean coupling
Source: Nat Commun. 2022 Apr 1;13:1730. doi: 10.1038/s41467-022-29392-4 (PMC8975866; doi:10.1038/s41467-022-29392-4)
Supplement: Supplementary file 1 — Supplementary Information [file 41467_2022_29392_MOESM1_ESM.pdf]

Supplementary information for ”the future poleward shift of Southern Hemisphere summer mid-latitude storm tracks stems from ocean coupling”

Rei Chemke[1]

[1] *Department of Earth and Planetary Sciences, Weizmann Institute of Science, Rehovot, Israel*

Supplementary Table 1. List of the 14 CMIP5 models analyzed in this study.

	Model	Modeling Center
1	bcc-csm1-1	Beijing Climate Center, China Meteorological Administration
2	BNU-ESM	College of Global Change and Earth System Science, Beijing Normal University
3	CanESM2	Canadian Centre for Climate Modelling and Analysis
4	CMCC-CMS	Centro Euro-Mediterraneo per I Cambiamenti Climatici
5	FGOALS-g2	LASG, Institute of Atmospheric Physics, Chinese Academy of Sciences; and CESS, Tsinghua University
6	FGOALS-s2	LASG, Institute of Atmospheric Physics, Chinese Academy of Sciences; and CESS, Tsinghua University
7	GFDL-CM3	Geophysical Fluid Dynamics Laboratory
8	GFDL-ESM2G	Geophysical Fluid Dynamics Laboratory
9	GFDL-ESM2M	Geophysical Fluid Dynamics Laboratory
10	IPSL-CM5A-LR	Institut Pierre-Simon Laplace
11	IPSL-CM5B-LR	Institut Pierre-Simon Laplace
12	MIROC-ESM	Japan Agency for Marine-Earth Science and Technology, Atmosphere and Ocean Research Institute (The University of Tokyo), and National Institute for Environmental Studies
13	MIROC-ESM-CHEM	Japan Agency for Marine-Earth Science and Technology, Atmosphere and Ocean Research Institute (The University of Tokyo), and National Institute for Environmental Studies
14	MPI-ESM-MR	Max Planck Institute for Meteorology (MPI-M)

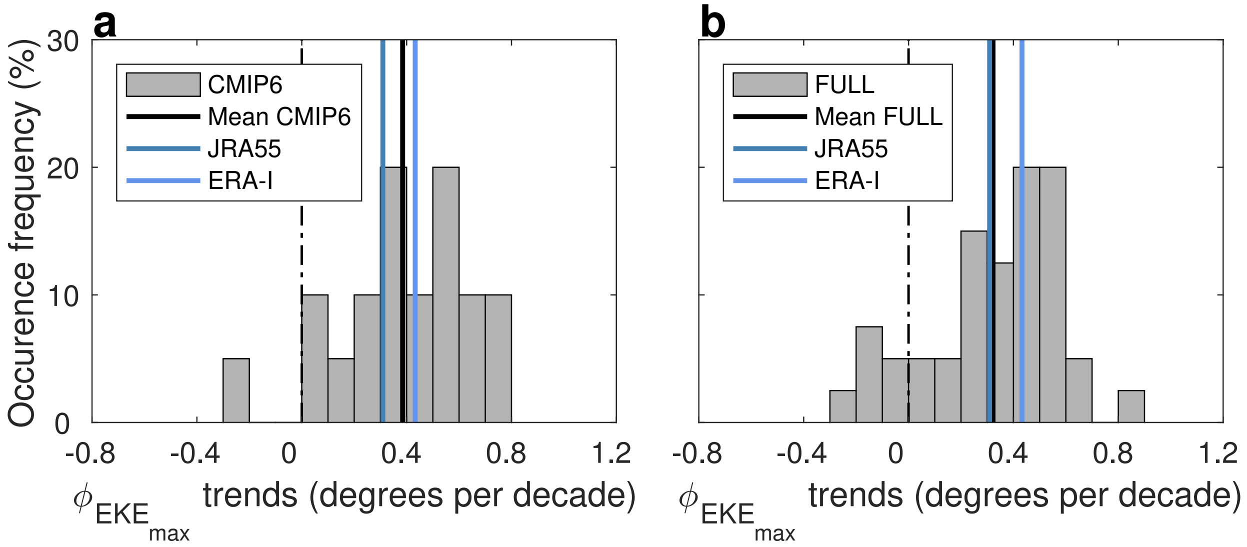

Supplementary Figure 1. The occurrence frequency (in percentage) of the 1979-2019 trends in the position of Southern Hemisphere mid-latitude DJF EKE ($\phi_{\mathrm{EKE_{max}}}$, degrees per decade) across **a,** CMIP5 models and **b,** FULL simulations. Black and blue vertical lines show the trends in the mean of the CMIP5/FULL ensembles and of the JRA55/ERA-Interim reanalyses, respectively.

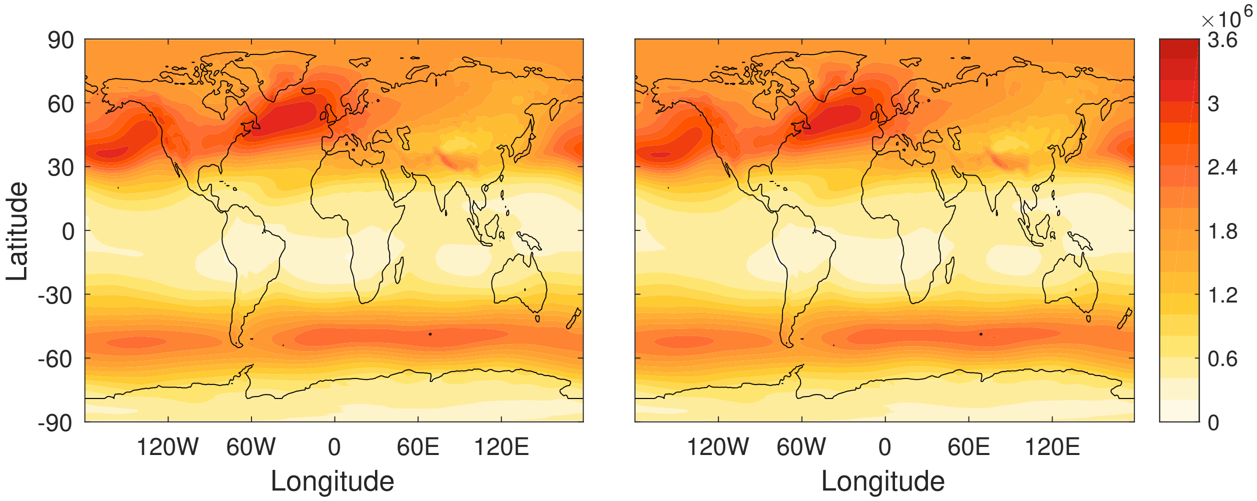

Supplementary Figure 2. Preindustrial climatology of DJF EKE ($\mathrm{Jm^{-2}}$) in the fully coupled (left) and slab ocean (right) models of the CESM1.

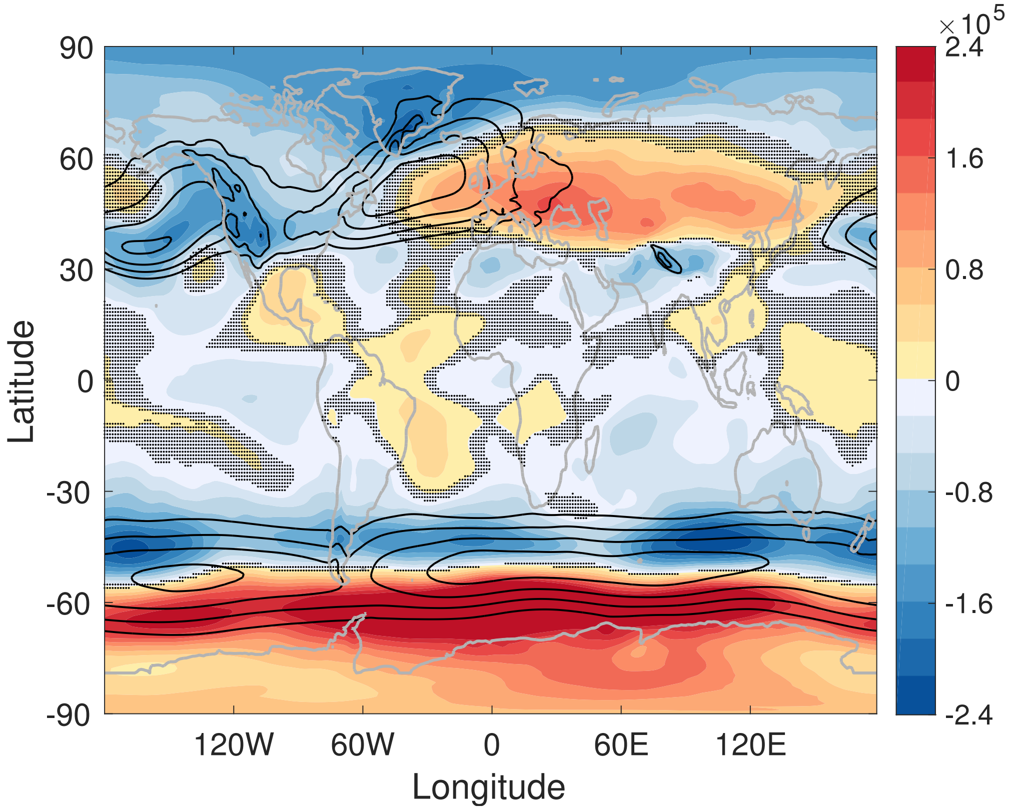

Supplementary Figure 3. The response to anthropogenic emissions of DJF EKE ($\mathrm{Jm^{-2}}$, colors) in the mean of the FULL ensemble. Black contours show the EKE distribution averaged over the 1980-1999 period in intervals of $3{\cdot}10^5$ $\mathrm{Jm^{-2}}$ and maximum values of $2.2{\cdot}10^6$ $\mathrm{Jm^{-2}}$ and $3{\cdot}10^6$ $\mathrm{Jm^{-2}}$ in the Southern and Northern Hemispheres, respectively. Black dots show where the response is not statistically significant at the 5% level based on a Student's t-test.

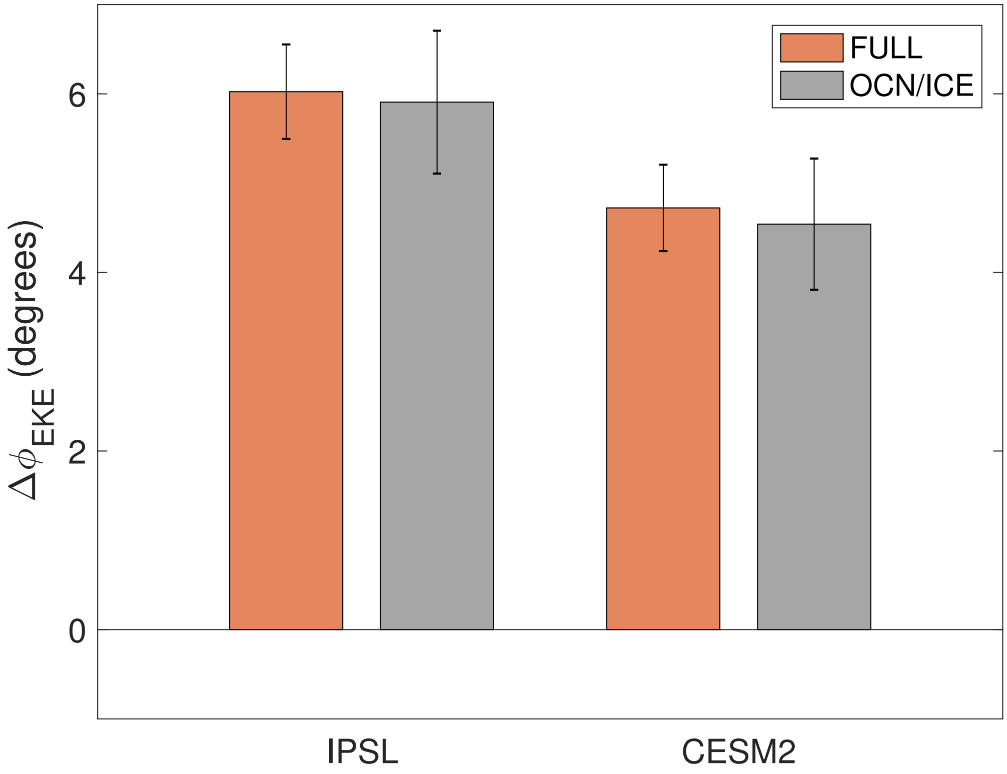

Supplementary Figure 4. The response to quadrupling of CO_2 concentrations, relative to preindustrial values, of the latitudinal position of EKE (degrees) in the fully-coupled versions of IPSL-CM6A-LR and CESM2 (red bars). The gray bars show the relative contribution to the EKE shift from ocean/sea-ice coupling (OCN/ICE). The error bars show the 95% confidence interval based on a Student's t-distribution.

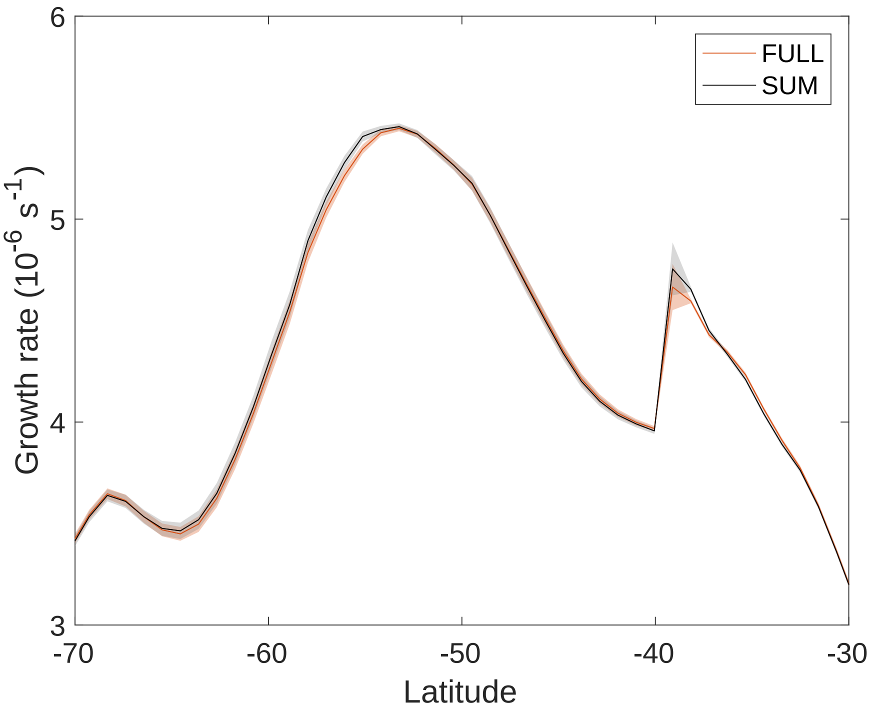

Supplementary Figure 5. The growth rate ($10^{-6}\,\mathrm{s}^{-1}$), averaged over the last 20 years of the 21st century, calculated from the linear normal mode instability analysis, as a function of latitude in the mean of the FULL ensemble (FULL, red line). Gray line shows the sum of the relative contribution to the 21st century growth rate from the mean zonal wind, static stability and tropopause height (SUM). Shading shows the 95% confidence interval based on a Student's t-distribution.

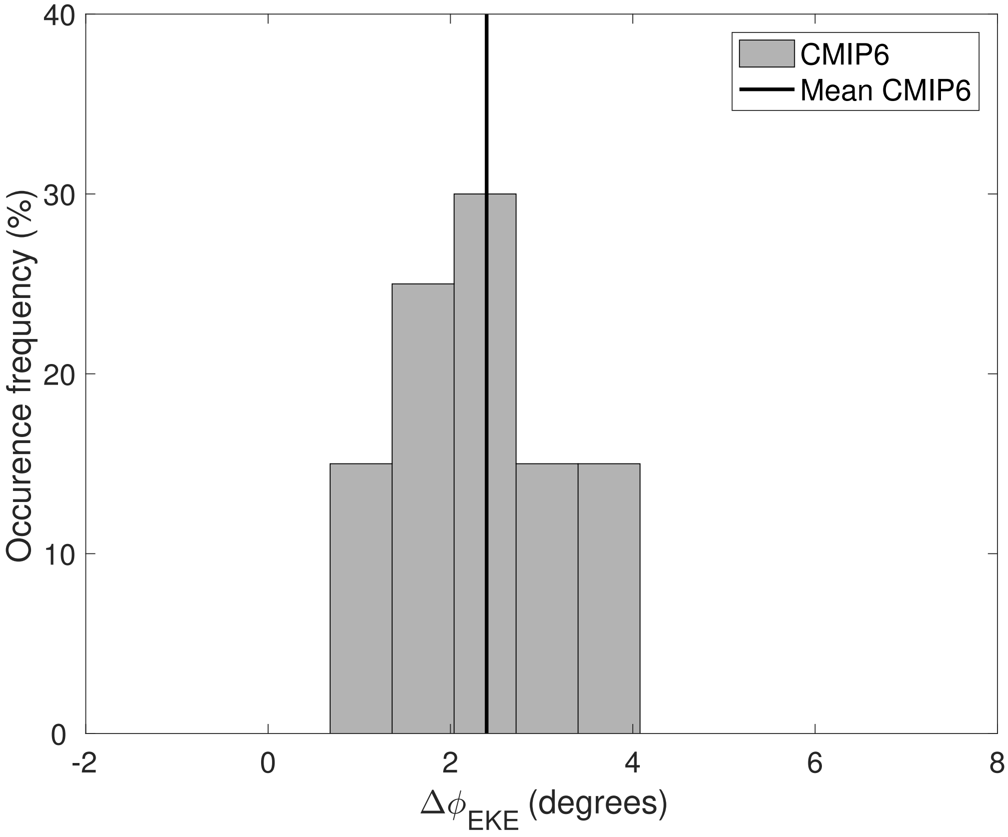

Supplementary Figure 6. The occurrence frequency (in percentage) of the poleward shift of Southern Hemisphere mid-latitude EKE ($\Delta\phi_{\mathrm{EKE}}$, degrees) across CMIP6 models (gray bars). Here the EKE is defined using a band-pass Butterworth filter of 2.5-6 days. The black vertical line shows the mean of the CMIP6 ensemble.

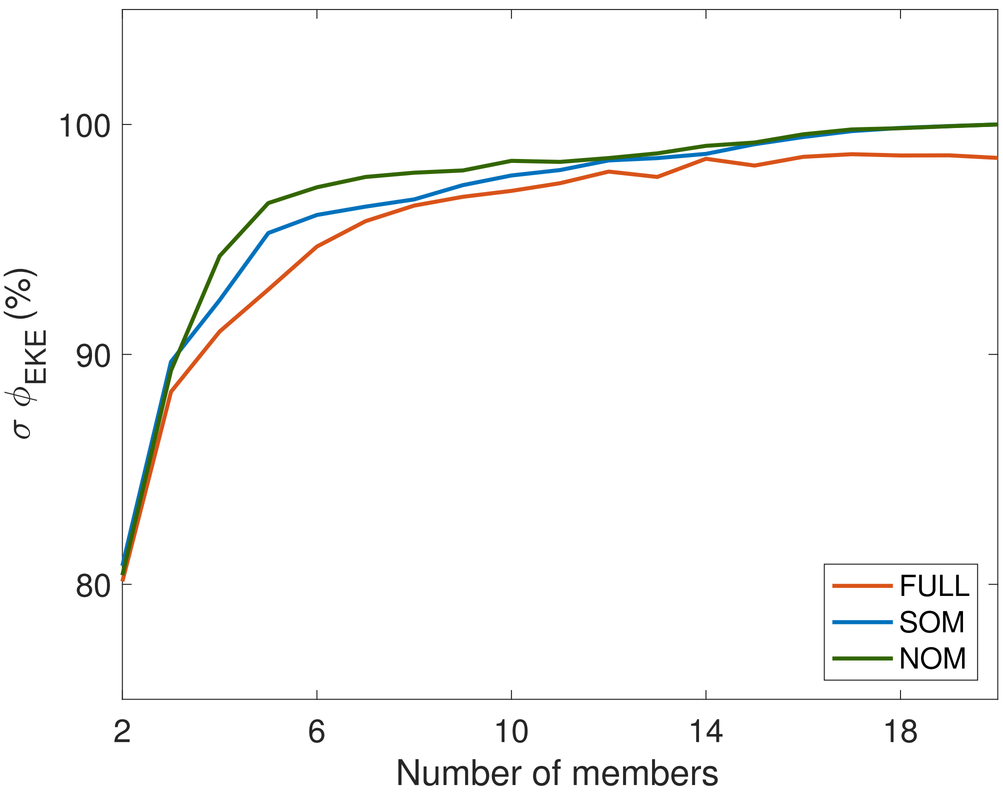

Supplementary Figure 7. One standard deviation of the EKE position across different number of ensemble members, relative to one standard deviation across all members. The standard deviation is calculated each year, and averaged over the 20th and 21st centuries, and over all combinations of number of ensemble members (or up to 1000 random combinations) in FULL (red), SOM (blue) and NOM (green).
